# Ulceration of the Legs

**Published:** 1855-05

**Authors:** Frederick G. Skye

**Affiliations:** Surgeon to the Hospital


*Ulceration of the Legs.* Clinical Lecture delivered at St. Bartholomew's Hospital, on Friday, Jan. 12th. By FREDERICK G. SKYE, Esq., F.R.S., Surgeon to the Hospital.

GENTLEMEN—I propose, in the ensuing lecture, to direct your attention to a group of cases that have recently come under treatment in the wards that have been placed under my charge by the treasurer of the hospital. They are those of chronic ulceration of the legs, and, for the most part, occurring in persons of advancing years. There are few persons attending the practice of our London hospitals who are not alive to the frequency of this form of disease. Our out-patients, as well as our dispensary receiving-rooms, teem with them. It may be truly said, their name is legion, whether in town or country. There is no disease so universal as the chronic ulcers of the leg in the lower classes of society, and, for reasons that I shall presently state, to this class are they almost exclusively confined. Eighteen years have elapsed since I published a small pamphlet on the treatment of this most common but troublesome malady. After eighteen years, a wider field of observation is open to me, and I have rejoiced in the opportunity of testing the truth of the views I then promulgated. They have been tried, and I am confirmed in their truth, and the result of that evidence I now proceed to place before you.

[The following cases are drawn up by Mr. Stanwell, Mr. Skye's house surgeon:]

---

W. G——, aged 41, admitted under Mr. Skye into Harley ward on Nov. 9th, 1854. On the left leg, on its inner aspect, was a large indolent ulcer, with very prominent white edges. He had been troubled with this for several years, and been unimproved by treatment. The ulcer was about the size of a crown piece. He took the common diets with a pint of porter daily, and soap-and-opium pill, five grains at night. The alteration which even two doses of the pill made in the appearance of the ulcer was as remarkable as it was beneficial. The granulations (before pale, flabby, and extremely unhealthy) were red, small, and covered with a thin layer of healthy pus; the edges, before unhealthy and white, were level with the granulations. The last change was, however, assisted by the application of strapping and bandage. He progressed favorably. The ulcer nearly healed, until Nov. 28th, when, with another patient in the ward, he was seized with phlebitis. The ulcer now rapidly lost ground. Under tonic and stimulating treatment, he again got better, and is now in Abernethy ward, almost convalescent.

---

G. C——, aged 59, was admitted into Harley ward on the 9th of Nov. 1854. He had suffered for many years from numerous small ulcers on the left leg and foot, for which, as a means of cure, and as a last resource, amputation was deemed advisable. The ulcers, on admission, presented a most unhealthy aspect; deeply excavated; white, overhanging margins; pale, flabby granulations, from which a thin, sanious discharge issued. On admission, he was ordered the common diet of the hospital, and a pint of porter daily. Ordered confection of bark two drachms, three times a day; acetate of morphia, two grains; cerate, a drachm, as an external application to the ulcers. This treatment he continued till the 27th of November, when he began to take the pills three times a day, the effect on the ulcers being manifestly very good. He is now lying in Abernethy ward, gradually getting better; under the same treatment.

---

J. P——, a healthy-looking countryman, was admitted into Harley ward on the 11th of November, 1854, suffering from a chronic ulcer of the left leg, which had for some years resisted all attempts to cure it. The man suffered a good deal frem cold extremities. The ulcer presented the ordinary characters of the indolent class. Good diet, rest, and the internal administration of opium, night and morning, in the form of soap-and opium pill, rapidly converted the unhealty granulations into red-pointed and cleanly secreting ones. After a short time, (on Nov. 30th,) he took half a grain of extract of opium, night and morning, as the combination with soap, seemed to relax the bowels. An attack of phlebitis, from which, under the treatment mentioned in the ward paper, (Dec. 1st,) he recovered, checked the process of cure (which was rapidly going on) for some days. On its cessation, the ulcer again assumed a healthy character, and he was discharged on the 4th of January.

---

Emily S——, aged four years, was admitted into Treasurer ward on the 10th of November, 1854. About an hour before a mail-cart had passed over her right foot and ankle, causing a severe and extensively lacerated wound of the dorsum and sole of the foot. In the former situation, the extensor brevis digitorum muscle, and the tendons of the extensors of the toes were exposed. The integument and fatty tissue on the sole were much lacerated The edges of the wound were brought into as accurate apposition as possible, and wet lint covered over the whole injured surface. The child progressed favorably, in every respect, until November 16th, when erysipelas (at the time very apparent) made its appearance, extending to the knee. The leg was wrapped in cotton wool, and a bread-and-water poultice applied to the foot. At the same time, she was ordered one grain of the disulphate of quinine every two hours, and six ounces of wine daily. The inflammation gradually subsided, the quinine having been previously increased to two grains, and afterwards changed for tincture of cinchona, forty minims, aromatic spirit of ammonia, twenty minims, every four hours. The granulations of the surface of the wound on Dec. 4th appearing indolent and unhealthy, Mr. Skye ordered one grain of soap-and-opium pill every night. This has been continued since Strapping has also been employed, and the child remains now in the hospital, a happy example of the beneficial effects of opium on unhealthy granulating surfaces.

---

Mr. P——, a London merchant, aged 66, tall and stout, had a chronic ulcer on his leg, nearly as large as the crown of a moderate sized hat. It had been his companion for seventeen years. He applied to two eminent surgeons, who said that they neither could nor dared to heal it. The supposed evil attending the arrest of a long-standing wound in the system you must take for what it is worth. My faith is somewhat equivocal. I told him I could cure it, and I dared to cure it. It discharged immense quantities of fœtid ichor, and had done so for years Mr P —— was almost excluded from the drawing-room of his friends. I gave him half a grain of extract of opium night and morning, and I strapped his ulcer daily. In three days he expressed astonishment at the altered aspect of his deeply excavated ulcer In a week I increased the dose to one grain. In forty-two days this immense wound had healed to the size of a small watch. His health improved under the treatment. During the above period he had taken no form of medicine but that I have mentioned.

Such is the treatment I have adopted in these cases of ulcer of the lower limbs; and you will naturally inquire into its principles and objects. for it is not sufficient that you learn the efficacy of a given remedy in any given disease. You are bound, as students, to inquire into the rationale or the principle of a remedy, that you may hereafter yourselves extend its application, and generalize on its influence, were it for no other purpose than that of comparing, classing, or distinguishing diseases; for it may not unreasonably be inferred that two or more apparently dissimilar diseases, which are amenable to the same remedy, must have at least some characters in common.

I venture to attribute to this remarkable drug the property of promoting the formation of healthy granulations on a surface that, notwithstanding all the previous appliances of surgery, is yet flat, pale, and ungranulating. Now, there is no example of the power of opium to effect this object more conclusive, or in which there is more work to be done, than that form of disease of which I am speaking, which consists of a gap formed on the surface of the body, of greater or less depth in diameter, and in which there exists not even a trace of a curative action; and yet the object is accomplished by means of this agent, and often with remarkable celerity. We call opium a stimulant and sedative. As a stimulant, it is not very often employed in practice; while its properties as a sedative are well known, and in daily requisition. Its property of mitigating pain and of promoting sleep is that for which it is almost exclusively employed; and so completely is its action associated with this sedative principle, that its occasional influence as a stimulant is almost entirely lost sight of, and the stimulating property has merged in the supposed sedative. How otherwise can we explain the reasoning of Mr. Pott, who may almost be termed the father of modern British Surgery? In speaking of the subject of opium in the treatment of gangrena senilis, he refers its undoubted efficacy to its property of soothing pain. He says: "I have always found that whatever tended to calm, to relax, and to appease, at least retarded mischief, if it did no more."

But, in truth, pain, though common, is by no means an invariable concomitant of senile gangrene; and it is tolerable notorious that opium is a valuable remedy in all cases of this disease. How then—on what principle does opium act in those numerous cases of senile gangrene that are destitute of pain, and in which it is an equally efficacious remedy?

I believe that its sedative properties have little concern with the result. In truth, opium is a most valuable stimulant of the vital powers, and whether its action originates with the centre or the periphery of the circulation, whether primarily on the heart or on the capillary vessels, I do not pretend to know. But there is no drug, simple or composite, known to our pharmacologists that possesses an equal power with opium, of giving energy to the capillary system of arteries, of promoting animal warmth, and thus maintaining an equable balance of the circulation throughout the body. To maintain the balance of the circulation! How much of meaning is attached to these words!—how many affections of the bodily frame may not be brought within the range of this definition! Take the common chilblain: what is it but a local congestion of blood caused by defective capillary power? There is no better remedy than opium; cold feet, as characterizing a person or a coustitution, equally relieved; senile gangrene, the result of arrest of the capillary circulation, or its apparent opposite, local hyperæmia—these diseases, one and all, manifest a local power, a failure in the balance of circulation. The term "inflammation," a word formerly in the mouths of our professional gentlemen on all occasions, is now limited in its application, and should be yet more limited, and I believe, in a yet more advanced state of medical science, will be restricted to an actually rare condition of the system. The influence of opium in such conditions is that of promoting a genial warmth over the system, a glow exactly resembling, and in fact identical with, that produced by the reaction on the system which is caused by the cold bath. *It is local health*, and the sensation is most agreeable. The benefit derived from opium, when administered for the purpose of arresting inflammatory action of the vessels, admits, I think, of much doubt, and should be resorted to with some hesitation as a remedial agent, though I am quite persuaded that the evil of its administration is greatly overrated. But who will profess ignorance in these days of the inestimable value of this agent when resorted to immediately after an attack of inflammation has been subdued by a local or general bleeding? Here we can imagine that the activity of the disease being checked, the diffusing influence of opium on the circulation may act as a simple derivative, operating on the vessels at the moment they are not indisposed to yield up their blood, and to which indeed they are compelled by the diffusive power of the general stimulus.

Many years ago, and before the introduction of railway traveling, I was summoned late one afternoon to see a patient some eighty miles from London. I traveled outside the mail. This occurred in the month of December, and the night was extremely cold. By some mistake, I omitted to bring my great-coat, and for the first hour I suffered a good deal. On reaching a town at some ten miles distance from London, I took the opportunity, while changing horses, to run across the street to a druggist's shop, where I ordered a draught, containing twenty-five drops of tincture of opium. I believe I was the only person outside the coach that night who did not suffer the slightest sensation from cold. But it will be urged by many, who have experimented on and who have observed less than I have done, the medical properties of opium, the infinite importance of studying the reactive effects of this deadly poison, and they would inquire into the condition of a person so treated on the following day. You may be assured that it amounts to *nil*. You will, I am sure, readily understand what I mean when I say the cold and the opium mutually balanced and mutually neutralized each other. There could be no action, because the influence of the depressing agent, viz., the cold, rather than otherwise, exceeded in duration that of the stimulant. If the period of prostration were brief, and limited to one or two hours, the argument might hold; but it is but a sorry objection to be urged after all.

I wish I could impress on the minds of the medical authorities in the Crimea the real benefit that might be derived to our noble troops, beaten down by intense cold and suffering in its various forms, from the judicious administration of opium. If twenty-five or thirty drops of tincture of opium, in addition to the ordinary quantum of rum, were administered to each soldier whose nightly services are required in the trenches or on guard, you would hear little complaint of cold for that night, neither would it produce the smallest tendency to sleep. And what do you imagine would be the objection urged against the proposition? "You would destroy the efficiency of the entire army; you would corrupt their morals; you would engender the most enervating habits; they would all degenerate into professed opium-eaters; and, in fact," says the alarmist, "the idea is preposterous."

Here again I assert that no possible evil could result; the only sensation, present and future, would be the absence of cold. If cold beget suffering, opium is the antidote of that suffering, and the one will assuredly neutralize the other.

Notwithstanding the prejudices and the bigotry that have long beset the public mind on this subject, and from which our profession is not totally exempt, there is no comparison to be drawn between the practice af dram-drinking, and the excessive indulgence in the use of opium. The man who indulges in spirituous liquors makes daily inroads on his digestive powers, not less than on his brain. His appetite is destroyed, and the pabulum for his blood is withheld from his circulation. He is stamped for life, and his perfect health is irrecoverable. The influence of opium, when taken as a means of indulgence, though deleterious, is not permanently injurious. It exercises no serious influence on his digestion, or on his cerebral organs, and the practice once controlled, leaves him in a condition to regain, without difficulty, the fullest vigor of both bodily and mental health.

I have related to you the particulars of several cases of chronic ulcer in which recovery was attributable to the medical properties of opium, and almost to opium alone. The character of these ulcers strongly marks the inactivity of their nature, and hence the class of society to which they belong. They are marked by a flat base, which indicates, by its pale, flabby uniformity of surface, that no reparatory action has approached it. It is often surrounded by a thick, high ridge of lymph, covered by unhealthy integuments. The depth of the ulcer, which may be seven or eight lines, is caused partly by the excavation of the ulcer below the natural level of the healthy integuments. So long as this ridge exists, although granulations may form, and will form from the date of the emp'oyment of the opium, yet cicatrization will never complete the process of cure unless some wound or ridge be absorbed. Now the action of opium is not alone exhibited in the development of healthy granulations, but in the entire complement of such actions as are required by the sore, viz., the formation of new material, and the absorption of the old.

The influence of the stimulant is exhibited, therefore, not on one particular function. It does not merely promote secretion, but it stimulates to healthy vital actions in their entirety, viz., secretion, organization, and absorption contemporaneously. The granulations are secreted and organized, while the circumvallation of unsound mateaial, the product of years of growth, is gradually absorbed, and reduced to the level of the surrounding integument. For the removal of this wall it is quite as indispensable to the ultimate result as the obliteration of the cavity by granulation. Without the two surfaces be brought to the same level, the process of cicatrization, or skinning over, will never be perfected.

If, therefore, we find that a disease like that I have described, and which exhibits so palpably a dormant condition of the remote capillaries, is amenable to this form of stimulant, which can only accomplish the cure by the substitution of healthy for morbid actions, why should we restrict its employment to this class of disease? Why, as I have elsewhere inquired, may we not experiment with success on any local disease dependent on the same cause—viz., an inert condition of the remote vascular system?

In claiming for opium the merit of rousing into healthy action the dormant capillary system, to the end of accomplishing the permanent cure of the chronic ulcer of the legs in old persons, I by no means wish you to infer that I consider all other modes of treatment unworthy of trial. Indeed I attach great value to that recommended by Mr. Baynton, of Bristol, and others; but, having tested their value, I have no hesitation in pronouncing that which I have recommended as by far the most certain and efficacious.—*London Lancet.*

